# Depressive Symptoms Correlate with Disability and Disease Course in Multiple Sclerosis Patients: An Italian Multi-Center Study Using the Beck Depression Inventory

**DOI:** 10.1371/journal.pone.0160261

**Published:** 2016-09-15

**Authors:** C. Solaro, E. Trabucco, A. Signori, V. Martinelli, M. Radaelli, D. Centonze, S. Rossi, M. G. Grasso, A. Clemenzi, S. Bonavita, A. D’Ambrosio, F. Patti, E. D’Amico, G. Cruccu, A. Truini

**Affiliations:** 1 Neurology Unit, Head and Neck Dept., ASL 3 “Genovese”, Genoa, Italy; 2 Dep. of Experimental Medicine, Section of Diagnostic Radiology, University of Genoa, Genoa, Italy; 3 Dept. of Health Sciences, Section of Biostatistics, University of Genoa, Genoa, Italy; 4 Dept. of Neurology, San Raffaele Scientific Institute, Milan, Italy; 5 Neurology Clinic, Dept. of Systems Medicine, Tor Vergata University, Rome & IRCCS Neuromed, Rome, Italy; 6 Neurology Clinic, Dept. of Systems Medicine, Tor Vergata University, Rome & Istituto Neurologico Mediterraneo Neuromed, IRCCS, Pozzilli, Rome, Italy; 7 Santa Lucia Foundation, IRCCS, Rome, Italy; 8 Neurology Clinic, Second University of Naples, Naples, Italy; 9 Department DANA, “GF Ingrassia”, Neuroscience Section (Multiple Sclerosis Centre), University of Catania, Catania, Italy; 10 Dept. of Neurology and Psychiatry, University of Rome—La Sapienza, Rome, Italy; Charite Universitatsmedizin Berlin, GERMANY

## Abstract

**Background:**

Depression occurs in about 50% of patients with multiple sclerosis. The aims of this study was to investigate the prevalence of depressive symptoms in a multicenter MS population using the Beck Depression Inventory II (BDI II) and to identify possible correlations between the BDI II score and demographic and clinical variables.

**Methods:**

Data were collected in a multi-center, cross-sectional study over a period of six months in six MS centers in Italy using BDI II.

**Results:**

1,011 MS patients participated in the study. 676 subjects were female, with a mean age of 34 years (SD 10.8), mean EDSS of 3.3 (0–8.5) and mean disease duration of 10.3 years (range 1–50 years). 668 (%) subjects scored lower than 14 on the BDI II and 343 (33.9%) scored greater than 14 (14 cut-off score). For patients with BDI>14 multivariate analysis showed a significant difference between EDSS and disease course. BDI II scores for subjects with secondary progressive (SP) MS were significantly different from primary progressive (PP) patients (p < 0.001) but similar to relapsing-remitting (RR) patients. Considering subjects with moderate to severe depressive symptoms (BDI II score from 20–63), in relation to disease course, 11.7% (83/710) had RR MS, 40.7% (96/236) SP and 13.6% (6/44) PP.

**Conclusions:**

Using the BDI II, 30% of the current sample had depressive symptoms. BDI II score correlates with disability and disease course, particularly in subjects with SP MS. The BDI II scale can be a useful tool in clinical practice to screen depressive symptoms in people with MS.

## Introduction

Multiple sclerosis (MS) is an inflammatory, demyelinating disease of the central nervous system (CNS) and results in a number of consequences, including psychological and psychiatric disorders. Depressive symptoms are reported in about 50% of people with MS [1].

The diagnosis of depression in MS is difficult particularly because several symptoms including fatigue, cognitive dysfunction and sleep disorders may confound the complex clinical picture [2]. It has also been shown that depressive symptoms are under-diagnosed in MS [3]. It remains unclear whether depressed mood associated with MS is due to disease awareness and/or represents a manifestation of neurobiological changes due to the disease itself [4,5]. A number of studies have linked MS-related depressive symptoms to several factors including autoimmune dysregulation [6], age, gender, stress, unemployment [7], endocrine functions [8], and MRI features including total lesion volume [9], regional T2 lesions [10], T1 lesions [11], as well as regional brain atrophy [11].

Studies evaluating the prevalence of depression vary in the methods used. Diagnostic interview, administrative data or scales have been used to detect depression [12]

Rating scales have been developed specifically for use in a medical/clinical setting and to facilitate the diagnosis of depressive symptoms, although there is no consensus on the optimal screening scale for depressive symptoms in people with MS. Several scales have been proposed to assess depressive symptoms in psychiatric settings such as the Hamilton Rating Scale for Depression (HRSD) [13], research diagnostic criteria (RDC) [14], Center for Epidemiological Studies Depression Rating Scale (CES-D)[15], Montgomery-Asberg Depression Rating Scale (MADRS) [16], Composite International Diagnostic Interview (CIDI) [17], Beck Depression Inventory II (BDI II) [18], Hospital Anxiety and Depression Scale (HADS) [19].

In MS literature, the CES-D has been used to evaluate the prevalence of depressive symptoms among people with MS in three studies [20–22]. Chwastiak [20] reported a frequency of depressive symptoms of 29% of 739 people with MS contacted through a postal survey. Patten [21] reported a prevalence of depressive symptoms of 25.7% of 163 subjects and Bamer [22] examined 530 people with MS from Eastern Washington and 138 subjects from Western Washington, with a prevalence of depressive symptoms respectively, of 51% and 45%. A lifetime prevalence of depressive symptoms of 22.8%, in a sample of 30 people with MS, was reported by Schiffer [23] using a psychiatric interview and the BDI. Joffe [24] and Minden [25] reported respectively, a lifetime prevalence of 42% in 100 people with MS and of 54% in 50 people with MS using the RDC. In a study of 221 people with MS, Sadovnick [26] reported a lifetime prevalence of 50% using a psychiatric interview and Patten [7] reported a lifetime prevalence of 22.8% in 136 people with MS using the CIDI.

A recent consensus of the American Academy of Neurology proposes the BDI as the preferable scale for screening for depressive symptoms MS [27]. Further, the BDI II has been shown to have good criterion validity for screening mood disorders with 85% sensitivity and 76% specificity in MS. [28].

The aims of this study were to investigate the prevalence of depressive symptoms in a multi-center MS population using the BDI II and to identify possible correlations between BDI II score and demographic and clinical variables.

## Methods

### Study Design

Data were collected in a multi-center, cross-sectional study involving six Italian MS centers, using a face-to-face structured questionnaire compiled by a neurologist. The questionnaire included demographic data, year of symptom onset and diagnosis, Expanded Disability Status Scale score (EDSS), clinical course, pharmacological therapies for MS and BDI II score. The only exclusion criteria was the inability to provide informed consent. All subjects enrolled had normal cognitive functioning as measured by the Mini-Mental State Examination (cut off > = 27). Only depressive symptoms present at the time of the interview were considered. All data were registered in an ad-hoc database. Subjects included in the study were at least 18 years of age, with a diagnosis of MS according to recognized criteria [29] or a clinically isolated syndrome (CIS), in a stable phase of the disease, with no relapses or worsening of more than 1 point on the EDSS in the previous three months.

Ethical approval for this study was obtained from the ethical committees of each participating MS center. Signed informed consent was obtained from each patient prior to enrollment in the study according to the Declaration of Helsinki.

### Assessment

The 21-item BDI II consists of four statements describing increasing intensities of depressive symptoms. It includes somatic and cognitive-affective symptoms and each item is divided into a scale from 0 to 3 reflecting the patient’s feelings in the previous two weeks. A total score from all 21 items is generated CS-you should add here what the possible score range is. A score of 14 or above is indicative of depressive symptoms [18]. Depressive symptoms was categorized as: minimal–moderate depressive symptoms (range 14–19), moderate–severe depressive symptoms (range 20–29), severe depressive symptoms (range 30–63) [30].

### Statistical Analyses

Means with standard deviations (SD) and ranges were shown for all quantitative characteristics. The BDI II score was categorized following a cut-off score of 14. Univariate logistic models were performed to assess which demographic and clinical characteristics were associated with depressive symptoms (BDI> = 14). Characteristics with a p-value lower than 0.20 were considered for the multivariate model with further adjustment for clinical center. Odds-ratios (OR) and 95% confidence intervals (CI) are reported. A p-value lower than 0.05 was considered statistically significant. SPSS (v.20; IBM Corp) was used for computation.

## Results

### Subjects

1,011 patients with MS participated in the study. 676 (66.9%) subjects were female and 335 were male (37.1%), with a mean age of 34 years (SD 10.8), mean EDSS of 3.3 (0–8.5) and mean disease duration of 10.3 years (range 1–50 years).

The majority of subjects had a relapsing-remitting (RR) disease course (n = 710, 70.2%), 236 (23.3%) had secondary progressive (SP) MS, 44 (4.4%) primary progressive (PP) MS and 21 (2.1%) had a clinically isolated syndrome (CIS). See Table 1.

**Table 1:** Clinical characteristics by subcategories of disease course.

Disease Course/n. patients (%)	Mean Age (years)	Mean Disease Duration (years)	Mean EDSS
RR–SM (70.2%)	31 (SD: 10.3)	8.2 (0–38 yrs)	2.2 (0–9)
SP–SM (23.3%)	36 (SD: 10.8)	17 (2–50)	6.5 (1–9.5)
PP–SM (4.4%)	43.7 (SD: 10.5)	10.6 (0–32)	6 (1–8.5)
CIS (2.1%)	30.7 (SD: 12.1)	2.5 (0–19)	1 (0–2.5)

n.- number, RR-relapsing-remitting, MS-multiple sclerosis, SD- standard deviation, SP-secondary progressive, PP- primary progressive, CIS- clinically isolated syndrome.

Regarding disease modifying treatment, 474 (46.9%) subjects were in therapy with interferon beta, 173 (17.1%) with glatiramer acetate, 43 (4.3%) with natalizumab and 12 (1.2%) with an immunosuppressive drug (methotrexate).

668 subjects (66.1%) scored lower than 14 on the BDI II and 343 subjects (33.9%) scored >14, indicating the presence of clinical depressive symptoms.

Of those subjects with a BDI II score of >14,158 (46%) scored in the minimally to moderately depressed category (BDI II range 14–19), 124 (36%) in the moderately—severe depressed category (BDI II range 20–29), and 31 (9%) in the severely depressed category (BDI II range 30–63).

A univariate analysis (Table 2 –left side) demonstrated that patients with a BDI II score >14 were older, with SP disease course, with a longer disease duration and higher EDSS score. There was no significant difference for gender.

**Table 2:** Distribution of demographic and clinical characteristics according to depressive symptoms.

Demographic and clinical characteristics		BDI II < 14 (n = 668)*	BDI II > = 14 (n = 343)*	Univariate OR (95% CI)	p-value	Multivariate OR (95% CI)	p-value
Age at diagnosis	Years	33.2 (10.4)	35.3 (11.3)	1.02 (1.01–1.03)	0.003	1.01 (0.99–1.02)	0.49
Gender	Male	232/335 (69.3)	103/335 (30.7)	1.00 (ref)	0.13	1.00 (ref)	0.067
	Female	436/676 (64.5)	240/676 (35.5)	1.24 (0.94–1.64)		1.34 (0.98–1.82)	
EDSS	Points	2.8 (2.1)	4.4 (2.5)	1.34 (1.27–1.42)	< 0.001	1.27 (1.15–1.41)	<0.001
Disease course	PP	34/44 (77.3)	10/44 (22.7)	1.00 (ref)	< 0.001	1.00 (ref)	0.007
	RR	521/710 (73.4)	189/710 (26.6)	1.23 (0.60–2.54)		3.31 (1.41–7.76)	
	SP	94/236 (39.8)	142/236 (60.2)	5.14 (2.42–10.89)		3.93 (1.77–8.74)	
	CIS	19/21 (85.7)	2/21 (14.3)	0.57 (0.14–2.32)		1.79 (0.38–8.38)	
Time from diagnosis of MS	Years	9.1 (6.9)	12.6 (9)	1.06 (1.04–1.08)	< 0.001	1.01 (0.99–1.03)	0.36

Ref: category of reference

*Values are reported as mean (SD) or as count/total count of category in row (%). In multivariate model ORs are adjusted also for center.

After multivariate analysis, the most relevant factors correlating with BDI score greater than 14 were EDSS and disease course (Table 2 –Right side).

Considering disease course, 189/710 (26.6%) RR patients, 142/236 (60.2%) SP,10/44 (22.7%) PP, and 2/21 (9.5%) CIS patients scored >14 on the BDI II. There was a significant difference between PP and RR subjects (p = 0.007) and between PP and SP (OR _SP vs PP_ = 3.93 (95% CI: 1.77–8.74; p < 0.001), when adjusted for EDSS. There was no significant difference between RR and SP (p = 0.52).

In the subgroup with BDI II score range 14–19, the mean BDI II score was 16 and mean EDSS score 3.9. One hundred and six out of 158 patients (67%) were RR, 46 (29.1%) were SP, 4 (2.5%) were PP and 2 subjects were CIS (1.3%).

In the subgroup with BDIII score range 20–29, the mean BDI II score was 23 and mean EDSS was 4.45. Sixty-seven out of 124 subjects (54%) were RR, 52 (41.9%) were SP and 5 (4.1%) were PP.

In the subgroup with BDIII score range 30–63, the mean BDI II score was 37 and mean EDSS 6.78. 26.2% (16/61) of subjects were RR, 72.1% (44/61) SP and 1.7% (1/61) PP. (Table 3)

**Table 3:** Score ranges of BDI II according to disease course.

Score ranges of BDI II for depressive symptoms subcategories	N° patients	Disease course	EDSS
Minimal–Moderate depressive symptoms (14–19)	158/343		3.9
		• RR = 106/158 (67.1%) • SP = 46/158 (29.1%) • PP = 4/158 (2.5%) • CIS = 2/158 (1.3%)	
Moderate–severe depressive symptoms (20–29)	124/343		4.45
		• RR = 67/124 (54%) • SP = 52/124 (41.9%) • PP = 5/124 (4.1%)	
Severe depressive symptoms (30–63)	61/343		6.78
		• RR = 16/61 (26.2%) • SP = 44/61 (72.1%) • PP = 1/61 (1.7%)	

Finally, considering subjects with BDI II score from 20–63, in relation to disease course, 11.7% (83/710) were RR, 40.7% (96/236) SP and 13.6% (6/44) PP.

## Discussion

The aim of the study was to evaluate the frequency of depressive symptoms in an unselected MS population using the BDI II scale and to evaluate whether depressive symptoms correlated with disease or demographic characteristics. Although there is no consensus about the optimal screening scale for depressive symptoms in MS, a recent consensus of the American Academy of Neurology states that the BDI II is the suggested scale to screen depressive symptoms in people with MS [27]. The scale is largely adopted in clinical practice and recently has been used to assess depressive symptoms as a secondary outcome in an MS clinical trial [31].

This is the largest study on depressive symptoms in MS patients, using a specific rating scale. A prevalence rate of 30% coincides with other reports on smaller samples [20,21,32]. On multivariate analysis, we found that EDSS and disease course are the most significant factors correlated with depressive symptoms The data reported by Chwastiak et al., using a postal questionnaire, are consistent with current results regarding the relationship to disability, but not with clinical course. Further, Chwastiak et al. found an inverse correlation with disease duration. On the other hand, Bamer found a correlation with EDSS and an inverse relation with disease duration but not with disease course. Comparing such results with the present study is difficult due to the differences in sample size, the scale utilized to identify mood disorders and clinical characteristics of the populations studied. For example in the Chwastiak paper, 51% of subjects had a RR course and mean EDSS score of 4.9, a more disabled population compared to the current sample. Depressive symptoms were twice as common in subjects with RR MS than in those with PP MS in a smaller sample size study (30 vs 106 patients), although subjects with SP MS were not included [33].

A mean BDI score difference between RR and SP MS patients was also found by Askari et al [34]. even if the authors reported only mean BDI score and not the number of MS subjects with BDI score greater than 14 and no data on BDI score in PP MS patients.

In this study, a significant difference was detected between PP and RR subjects (p = 0.007) and between PP and SP (p < 0.001) when adjusted for disease course, while not between RR and SP. Disease course was the key factor related to the frequency of pathologic BDI II score and when PP and SP subjects with similar EDSS scores were compared (6 vs 6.04), the frequency of depressive symptoms (BDI II > = 14) significantly differed (22.7% in PP and 60.2% in SP patients).

Disease course was also relevant when correlated with BDI II scores subcategories, in which the majority of subjects with moderate to severe depressive symptoms (BDI II 20–63) had a SP disease course (40.7% vs. 11.7% RR and 13.6% PP). This finding, in our opinion, is particularly important given the clinical and practical implications considering that more than half of subjects with SP MS experience clinical depressive symptoms, frequently moderate to severe.

One possible explanation of this finding is the correlation between brain damage and the frequency of depressive symptoms, more important than disability per se. Subjects with SP and PP MS had similar EDSS scores although in MS disability is determined by a combination of cerebral and spinal cord lesions and brain lesion load is usually higher in patients with a SP disease course than in PP MS [35–36], Given this, it is possible to speculate that disability in subjects with a PP course is more related to spinal cord damage rather than cortical-subcortical lesions. On the other hand, correlation of several brain MRI metrics and depressive symptoms well documented and signifies that the more brain damage detected, more likely is the probability for the presence of depressive symptoms. Such a hypothesis is consistent with other reports that found less profound cognitive dysfunction in PP patients versus SP subjects [37].

The current study presents some limitations. The aim of the study was to report the frequency of depressive symptoms in MS patients in an out-patient setting, thus cognitive dysfunction and fatigue, factors that can have a role in the frequency of “pathological” BDI II scores, were not investigated.

Although a good correlation between the BDI and DSM IV criteria for depression has been found diseases other than MS such Parkinson’s [38].

In our study depressive symptoms were not classified following the DSM-IV and we are aware that as stated in a recent review “self-report rating scale are useful for quickly capturing an array of symptoms, they cannot be used to make a formal diagnosis of any mood disorder”. [39].

The BDI does not assess all of the symptoms covered by DSM-IV criteria, and we are aware that the clinical use of the BDI is primarily to assess the severity of depressive symptoms in patients with a previously diagnosed depressive illness. In neurological diseases, such as MS, excluding somatic symptoms in the diagnosis of depression may reduce the rate of diagnosis. [40] On the other hand to use somatic symptoms of BDI score to measure the severity of depression in MS patients may lead to elevated score translating in higher estimates of depression when symptomatology is related to MS itself [41]. Having in mind such limitation the use of the BDI in clinical practice should help neurologists, who are not practiced with the DSM-IV, to flag potentially depressed patients for a more specific evaluation. From a clinical point of view over-reporting of depressive symptoms may not have more serious consequences other than a higher number of patients referred for psychiatric follow-up.

The study, to our knowledge, is the largest reported in the literature using a specific rating scale and underlines the importance of screening for depressive symptoms in patients with MS in clinical practice.

Considering the high frequency of depressive symptoms in MS, the serious implications on an individual’s quality of life, and the possibility for successful treatment, the BDI II could be used in clinical practice as a screening tool for depressive symptoms in people with MS. Clinicians should be particularly vigilant concerning patients with a SP disease course because these individuals may be particularly at risk for developing significant depressive symptoms.

## Supporting Information

S1 File Data set. (XLSX)
